# Liquid- and Gas-Phase Diffusion of Ferrocene in Thin Films of Metal-Organic Frameworks

**DOI:** 10.3390/ma8063767

**Published:** 2015-06-19

**Authors:** Wencai Zhou, Christof Wöll, Lars Heinke

**Affiliations:** Institut für Funktionelle Grenzflächen (IFG), Karlsruher Institut für Technologie (KIT), Hermann-von-Helmholtz-Platz 1, Eggenstein-Leopoldshafen 76344, Germany; E-Mails: wencai.zhou@partner.kit.edu (W.Z.); christof.woell@kit.edu (C.W.)

**Keywords:** metal-organic frameworks, diffusion, mass transfer, gas phase, liquid phase

## Abstract

The mass transfer of the guest molecules in nanoporous host materials, in particular in metal-organic frameworks (MOFs), is among the crucial features of their applications. By using thin surface-mounted MOF films in combination with a quartz crystal microbalance (QCM), the diffusion of ferrocene vapor and of ethanolic and hexanic ferrocene solution in HKUST-1 was investigated. For the first time, liquid- and gas-phase diffusion in MOFs was compared directly in the identical sample. The diffusion coefficients are in the same order of magnitude (~10^−16^ m^2^·s^−1^), whereas the diffusion coefficient of ferrocene in the empty framework is roughly 3-times smaller than in the MOF which is filled with ethanol or *n*-hexane.

## 1. Introduction

Metal-organic frameworks (MOFs) are crystalline, nanoporous hybrid materials, which attract a considerable amount of attention due to their unique properties, such as their very large specific surface area and their enormous variety [1,2]. In addition to this, MOF properties can be varied over wide ranges in a rational manner, resulting in tunable structures. This enables many interesting applications, such as the storage and separation of gases, as sensors as well as in catalysis [3,4,5]. For all these applications, the interaction of the guest molecules in the pores with the framework is crucial. Therefore, the transport properties of the guest molecules are among the key properties of MOFs. The diffusion of various gas molecules in different MOF structures has been intensively studied, e.g., references [6,7,8,9,10]. On the other hand, the diffusion of the guest molecules from the liquid phase has only been investigated in a few publications [11,12,13]. So far, a comparison of the mass transfer properties in the MOF pores in the liquid and the gas phase is lacking.

**Figure 1:**
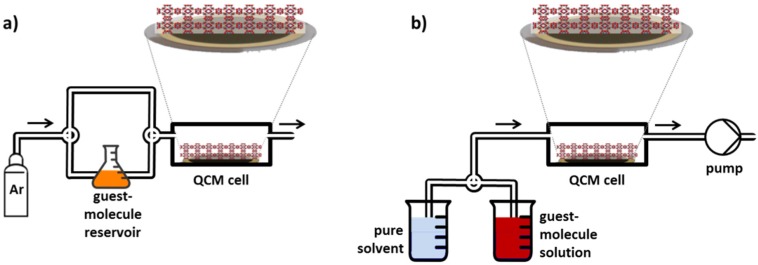
Setup of uptake experiments from gas and liquid phase. (**a**) A gas flow system where the carrier gas (here: argon) flows through the quartz crystal microbalance (QCM) cell, *i.e.*, over the QCM sensor, is used for uptake experiments from the gas phase. The carrier gas flow can be enriched with the vapor of the guest molecules by passing through (or over) the reservoir of the guest molecules; (**b**) The uptake of guest molecules from the liquid phase can be investigated by the same sample by connecting the QCM cell to a peristaltic pump, so that either the pure solvent or the guest-molecule solution can be pumped through the QCM cell. Switching from the pure gas flow, or pure solvent, to the enriched gas flow, or solution, results in the uptake of the guest molecules by the surface-mounted metal-organic framework (SURMOF). The mass changes are recorded by QCM.

Here, we used thin films of MOFs in combination with a quartz crystal microbalance (QCM) to investigate the uptake from the liquid and from the gas phase. The MOF films are prepared on a QCM sensor in a layer-by-layer fashion employing liquid-phase epitaxy. These thin MOF films are referred to as surface-mounted MOFs, SURMOFs [14,15,16]. Various surfaces can be modified to direct and improve the SURMOF growth on top, e.g., several metal surfaces can be functionalized with a self-assembled monolayer (SAM) [17] of organic molecules with a thiol anchor group and a functional head group. The SURMOF crystal orientation is determined by the functionalization of the substrate surface [18], and their thickness can be directly controlled by the number of synthesis cycles. The SURMOF morphology is usually smooth and homogenous [19] and even transparent thin films can be prepared [20]. In addition to this, the SURMOF growth process may hinder the interpenetration of the MOF [21] and it enables hetero-epitaxy, *i.e.*, MOF-on-MOF [22,23]. SURMOF films in combination with QCM were used to investigate the diffusion of vapor molecules such as pyridine [24], cyclohexane [25] or ferrocene [26] in MOFs. On the other hand, the QCM was also used to investigate the SURMOF growth in the (alternating) synthesis solutions, *i.e.*, in the liquid phase [27]. Here, we combine both approaches and investigate the uptake of a probe molecule, ferrocene, from the gas phase, *i.e.*, from ferrocene vapor in a carrier gas, and from the liquid phase, *i.e.*, from ethanolic or hexanic ferrocene solutions, by the identical SURMOF sample of type HKUST-1, see Figure 1.

## 2. Experimental Section

**Figure 2:**
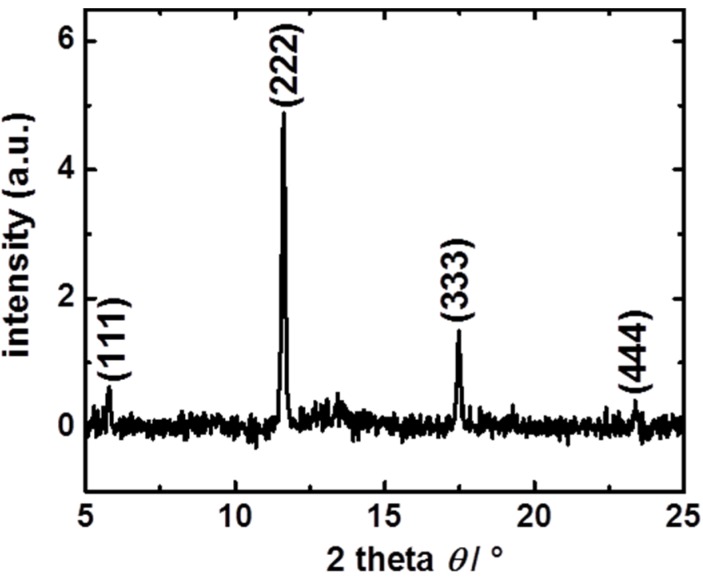
X-ray diffractogram of the HKUST-1 SURMOF. The diffraction reflexes at 5.8° (111), 11.6° (222), 17.5° (333) and 23.3° (444) demonstrate the crystallinity of the HKUST-1 SURMOF as well as the oriented growth in [111] direction. The X-ray diffractogram was measured with X-ray radiation of 0.154 nm wavelength by using an out-of-plane X-ray diffractometer of type Bruker D8 Advance.

For recording the mass changes and the uptake of the guest molecules by the SURMOF, a QCM from Q-Sense is used, which works at a resonance frequency of about 5 MHz. The investigated SURMOFs of type HKUST-1 [28] were prepared on gold-coated QCM sensors, whose surfaces were modified with an 11-mercaptoundecanol (MUD) SAM, in a layer-by-layer fashion by using the spray method [29]. By using the spray method, which enables upscaling to coating large areas, the ethanolic solutions of both MOF-components, *i.e.*, of the metal complexes (copper (ii) acetate) and of the organic linker molecules (1,3,5-benzenetricarboxylic acid), are alternatively sprayed on the substrate surface. The X-ray diffractogram of the prepared HKUST-1 SURMOF shows crystalline oriented growth, see Figure 2.

Mass changes of the sample on the QCM sensor can be determined by the Sauerbrey equation [30], Δ*m* = *C* × Δ*f*_n_/*n*, with Δ*m* denoting the mass change, *C* the Sauerbrey constant (here: 17.7 ng·cm^−2^·Hz^−1^) and *n* the overtone number. The sample thickness was determined from the change of the resonance frequency *f*_1_ of the QCM sensor before (4,952,150 Hz) and after (4,951,556 Hz) the SURMOF synthesis. A SURMOF mass of 10.5 µg·cm^−2^ follows, which corresponds to a SURMOF thickness of about 100 nm.

The uptake of ferrocene from the gas phase is performed by connecting the QCM cell to a gas flow system [26]. The flow of the pure carrier gas (argon with a flow rate of 10 mL·min^−1^) is instantly switched to a flow passing over the compound of the guest molecules (here: ferrocene powder with a saturation vapor pressure of about 1 Pa at room temperature [31]) resulting in a gas flow enriched with the vapor of the guest molecules. When the enriched gas flow passes through the QCM cell, the guest molecules are diffusing into the SURMOF and the uptake can be studied by the mass change of the SURMOF on the QCM sensor. It should be noted that the concentration of argon in MOFs of type HKUST-1 at room temperature and a pressure of about 1 bar is very small [32]. Therefore, the argon molecules in the pores as well as the diffusion of argon during the uptake of the guest molecules can be neglected and the activated MOF pores can be considered as empty.

The uptake from the liquid phase is investigated by initially pumping the pure solvent (*i.e.*, ethanol or *n*-hexane) through the QCM cell. Then, the solvent is instantly switched to the respective ferrocene solution. Here, ferrocene concentrations were chosen which correspond to 90% of the saturation solubilities [33], *i.e.*, 90 mM ferrocene in ethanol and 155 mM ferrocene in *n*-hexane. To ensure that the uptake by the SURMOF is controlled by diffusion in the nanopores and not by convection through the QCM cell, the flow rates of the gases and liquids through the QCM cell need to be large enough Here, it was set 10 mL·min^−1^ for the gas flow and roughly 1 mL·min^−1^ for the liquid phase.

**Figure 3:**
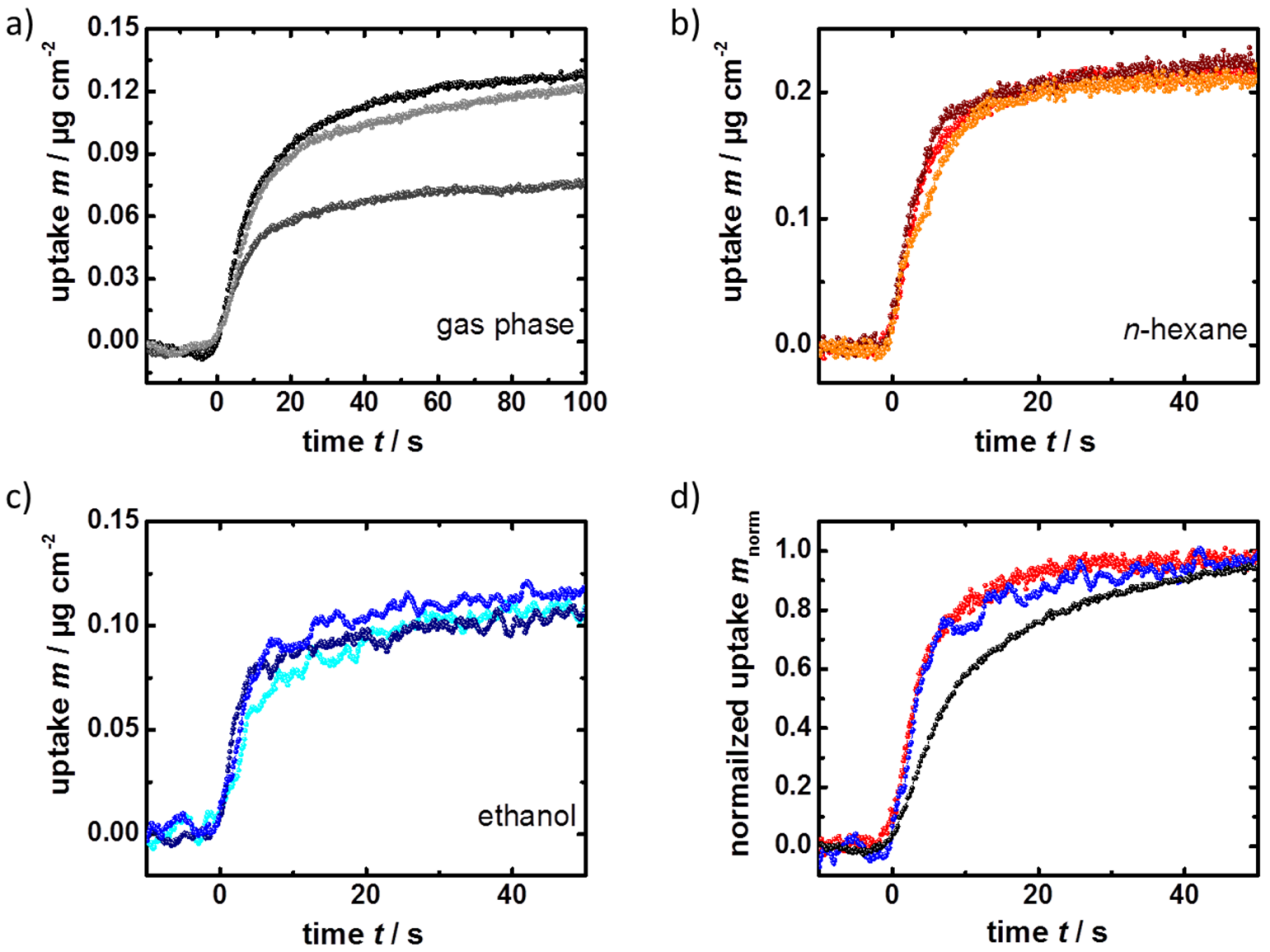
Ferrocene uptake by HKUST-1. The uptake curves of ferrocene vapor in argon carrier gas (**a**); of ferrocene in *n*-hexane (**b**); and of ferrocene in ethanol (**c**); are measured by QCM. For comparison of the uptake kinetics, the normalized uptake by the SURMOF from the liquid and the gas phase are shown in (**d**). The black sphere show the uptake of the ferrocene vapor, red of the *n*-hexanic ferrocene solution and blue of the ethanolic ferrocene solution.

To increase the significance of the data and to exclude artifacts, the uptake of the guest molecules from the liquid and the gas phase are alternatively repeated. The uptake curves of the ferrocene by the SURMOF are shown in Figure 3. All experiments were performed at a temperature of 25 °C. Before each experiment, the sample was activated in a flow of pure argon at 65 °C for several hours, to guarantee that no molecules, such as water, were adsorbed in the pores and to guarantee reproducible results.

## 3. Results and Discussions

The uptake curves recorded by QCM enable an analysis of the equilibrium concentration and of the mass transfer parameter, *i.e.*, the diffusion coefficient. Since the SURMOF pores which are activated in argon can be considered as empty, the QCM experiments allow a straightforward quantification of the uptake amount, as demonstrated in references [34,35,36]. The ferrocene uptake is determined to be 0.11 ± 0.02 µg·cm^−2^, which corresponds to roughly 3/4 ferrocene compounds per HKUST-1 unit cell. Since ferrocene is a solid which slowly sublimates, it can be assumed that the obtained partial pressure of ferrocene is (significantly) smaller than the saturated vapor pressure of ferrocene, resulting in an equilibrium concentration which is (much) smaller than the maximum ferrocene concentration in the MOF pores.

The uptake from the liquid phase is more complex. The pores are initially filled with the solvent. When the investigated guest molecules are diffusing into the pores, the solvent molecules are displaced. Therefore, the determination of the uptake amount is not straightforward and requires the knowledge of the densities of the solvent in the MOF pores, with and without ferrocene. Due to the fact that the mass change is proportional to the ferrocene uptake, the recorded uptake curves can be used to investigate the kinetic properties and to determine the diffusion coefficient of the ferrocene compound in solution.

**Figure 4:**
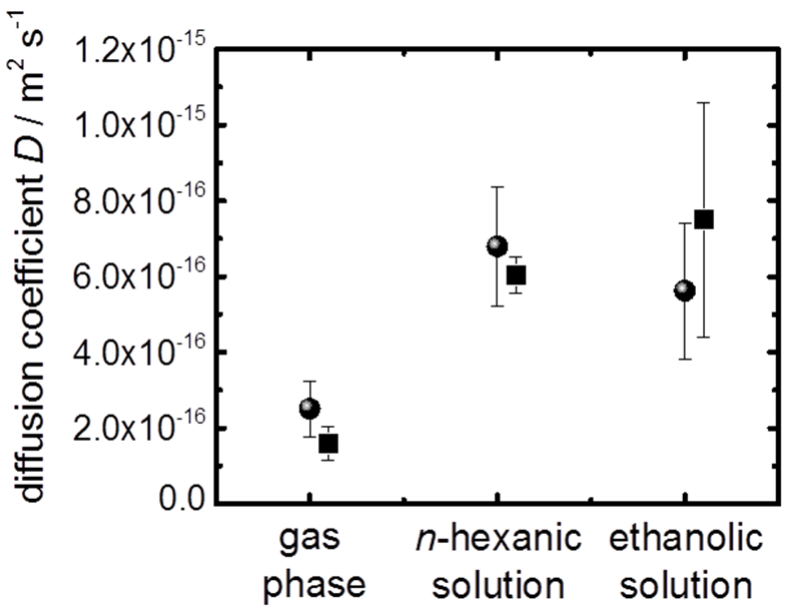
Diffusion coefficients of ferrocene in HKUST-1. The average diffusion coefficients with the respective standard deviation are shown for the ferrocene vapor in the argon carrier gas, for ferrocene in *n*-hexanic solution and for ferrocene in ethanolic solution. The results of both samples are shown; spheres: sample 1, squares: sample 2.

It was determined from the uptake curves (Figure 3) that the ferrocene uptake from the gas phase proceeds with an average time constant *τ* of 13 ± 4 s, from the *n*-hexanic solution with a time constant of 4.9 ± 1.1 s and from the ethanolic solutions with a time constant of 5.8 ± 1.9 s. The error denotes the standard deviation of the experimental results. By using the relationship *D* = *l*^2^/(3*τ*) (reference [37]) and using the determined SURMOF thickness *l* of about 100 nm, the diffusion coefficient *D* for ferrocene in the (initially) empty MOF, *i.e.*, of ferrocene during the uptake from the gas phase, was determined to be (2.5 ± 0.7) × 10^−16^ m^2^·s^−1^. For the uptake from the liquid phase, ferrocene diffusion coefficients of (6.8 ± 1.6) × 10^−16^ m^2^·s^−1^ in the *n*-hexanic solution and (5.6 ± 1.8) × 10^−16^ m^2^·s^−1^ in the ethanolic solution were calculated. These experimental findings were reproduced by an HKUST-1 SURMOF of 110 nm thickness (sample 2), where diffusion coefficients of (1.6 ± 0.5) × 10^−16^ m^2^·s^−1^ from the gas phase, (6.0 ± 0.5) × 10^−16^ m^2^·s^−1^ from the hexanic solution and (7.5 ± 3.1) × 10^−16^ m^2^·s^−1^ from the ethanolic solution were determined, see Figure 4.

This means that the uptake from the ferrocene solution by the solvent-filled HKUST-1 proceeds roughly 3-times faster than the uptake of the ferrocene vapor by the empty HKUST-1 framework. This is remarkable, since the solvent molecules have to leave the pores to allow the ferrocene to enter the solvent-filled MOF pores. It may be assumed that the fast diffusion in the solvent is caused by the interaction of the ethanol or *n*-hexane solvent molecules, which occupy attractive adsorption sites in the MOF framework. Thus, the ferrocene molecules are less strongly adsorbed in the pores and can diffuse faster through the framework. It can be assumed that the diffusion of small molecules like ethanol and *n*-hexane is much faster than of ferrocene. Therefore, the counter diffusion of the solvent molecules does not slow down the ferrocene uptake.

A similar behavior was found in reference [38], where the self-diffusion coefficient of methanol in MOFs of type ZIF-8 was found to be larger than the transport diffusion coefficient. The reason for this anomaly is the strong interaction between the methanol molecules and the “intercage hopping” diffusion process in the ZIF-8 framework.

The pore windows of HKUST-1 have a diameter of roughly 0.9 nm, which is significantly larger than ferrocene with a kinetic diameter of about 0.66 nm [39]. In a previous study, the uptake of ferrocene by MOFs of type Cu_2_(ndc)_2_(dabco) with pore window diameters of 0.57 nm was investigated [26]. There, the diffusion coefficient of ferrocene is three orders of magnitude smaller than in HKUST‑1. This shows that the pore size has a dramatic impact of the diffusion properties, especially if the pore diameter and the guest molecules have a comparable size.

It should be noted that, due to the fact that the pristine samples were used directly after the synthesis, surface barriers [40,41,42], which may hinder the uptake and release of the guest molecules, are neglected during the analysis of the data [25].

## 4. Conclusions

The diffusion of ferrocene in MOFs of type HKUST-1 was investigated by using thin MOF films in combination with QCM. For the first time, the diffusion of the guest molecules in the gas phase, where the guest molecules are diffusing into an initially empty framework, and in the liquid phase, where the guest molecules are diffusing into a solvent-filled framework, are compared. It was found that in the MOF, the diffusion coefficient of ferrocene in the liquid- and in the gas-phase are in the same order of magnitude; with a ferrocene diffusion coefficient which is roughly 3-times smaller in the empty MOF (*i.e.*, uptake from the gas phase) than in the liquid-filled MOF (*i.e.*, uptake from the ferrocene solutions). It may be assumed that other liquids may have a larger impact on the diffusion and significantly slow down or accelerate the ferrocene uptake.

Due to their general applicability, SURMOF films in combination with QCM show great potential for investigating the diffusion of guest molecules in MOFs. Not only the uptake from the gas and from the liquid phase can be investigated, but also guest-host systems with diffusion coefficients ranging over at least six orders of magnitude can be studied. So far, mass transfers with diffusion coefficients in the range of 6 × 10^−19^ m^2^·s^−1^ [26] to 6 × 10^−13^ m^2^·s^−1^ [25] have been investigated. It might be assumed that even slower or faster diffusion processes can be investigated, for instance by fully utilizing the time resolution and by increasing or decreasing the SURMOF thickness.
